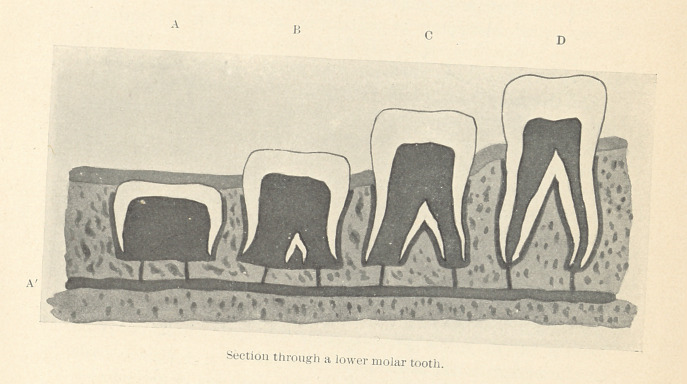# The Dental Pulp, Viewed without the Microscope

**Published:** 1903-06

**Authors:** Thos. E. Constant

**Affiliations:** Scarborough, England


### THE DENTAL PULP, VIEWED WITHOUT THE MICROSCOPE.[1]

BY THOS. E. CONSTANT, M.R.C.S., ETC., SCARBOROUGH, ENGLAND.

Before entering upon the subject-matter of the present paper may I be allowed to express my appreciation of the honor conferred upon me by the invitation of the Council of the American Medical Association, an Association which enrolls in its list of membership names which are known to and revered by scientists all the world over. The invitation was not accepted without diffidence, for the subject allotted is a narrow one and the investigators many and devoted; indeed, it may safely be affirmed that during the last decade no structure in the human body has been more often or more closely scrutinized than the dental pulp. The majority of the observers have, however, given most attention to those points which require the microscope for their elucidation, and it is partly on that account that I intend to-day to confine myself to the macroscopic aspect of the subject.

The human dental pulp has been arbitrarily defined by an American writer as commencing during the fourth month of the fetal existence. Prior to that time it is the "dental papilla." This distinction is convenient, and, although I am ignorant as to whether

---

[1] Abstract of a paper read before the American Medical Association, Section on Stomatology, New Orleans, May 5 to 8, 1903.

it has met with general acceptance in this country, for the purposes of the present paper I shall adopt it.

At the end of the fourth month of fetal existence dissection reveals the pulps of the milk-teeth as translucent gelatinous substances, which roughly correspond in shape to the crowns of the teeth which are respectively formed from them. They are generally described as lying in a shallow groove or gutter of bone which is all that exists at that period of what is later known as the alveolar portion of the jaws. The pulps are severally surrounded by a membrane to which they are not anywhere adherent except at their bases. Between the investing membrane and the pulp there is always a fluid which John Hunter likened to the synovial fluid in joints. In the case of the lower animals this fluid is often found in considerable quantity. The membrane is, of course, the tooth-sac. What the fluid is I do not know. By stripping the periosteum from the jaws the tooth-sacs and their contents can be removed entire from the gutter of bone in which they lie. At this period (fourth to fifth month), when the dental sacs have been thus removed the groove in which they lie is found to be traversed in the incisor region by slight ridges, which form the commencement of the alveolar septa between the incisor teeth. On opening the tooth-sacs they are found to be adherent below to the tooth-pulps and above to blend indistinguishably with the oral mucous membrane. The apices of the pulps show signs of commencing calcification, the incisors being tipped with little caps of bony material. As calcification proceeds these caps gradually increase in thickness and extend downward over the sides of the pulps, and at the end of nine months of fetal existence the central incisors are about two-thirds calcified; that is to say, the edge of the calcified caps almost reaches the bony floor upon which the pulp rests. In the mean time the bony gutter has undergone a marked transformation. Its edges have grown up around the pulps and the transverse ridges already mentioned have also grown up, forming complete septa between the developing incisor teeth. In the same way the pulps of the developing cuspid and molar teeth have been enclosed, bony septa of which there was no indication at four months having sprung up. Thus each developing tooth has become enclosed in a separate crypt, the floor of which is formed by the original gutter of bone, the labial and lingual walls by the upgrowth of its edges, and the mesial and distal by the upgrowth of the bony septa. Most of the text-books state that it is about this time (nine fetal months) that a roofing in of the crypts takes place by an arching over of the walls. Thus Broomell writes in describing the development of the crypts:

"Beginning with a simple groove, or gutter, into which the tooth follicles hang, the follicles exerting a controlling influence over its form. Next comes the appearance of septa between the anterior follicles, which at this period are somewhat irregularly placed in the arch, followed in a few weeks by a well-defined partition between the cuspids and molars, until finally, at birth, each follicle is enclosed in its individual crypt, with the single exception of the second molar, in which the distal septum, or that which is to separate it from the first permanent molar, has not yet made its appearance. As the tooth-follicles increase in size, by the development of the teeth within, they become more perfectly enclosed in the bony vaults, the sides of the alveolar walls arching over and almost completely enclosing the developing teeth."

Tomes, speaking of the condition of the mandible at the time of birth, quotes: "The alveolar margins are deeply indented with large open crypts." As a matter of fact, both of these descriptions are incorrect, for in the nine months' fœtus and at birth the crypts are completely closed. It is true that the bone which forms the roof of the crypts is very thin and parchment-like, but it is always there. Broomell is wrong, too, in his description in so far that he makes it appear that the roof is formed very late and by an arching over of the walls. The roof, such as it is, is complete before the bony septa between the various teeth, and what is usually described as a groove or gutter is really a tunnel with a very thin bony roof. There are one or two specimens in the museum of the Odontological Society of Great Britain which illustrate this very clearly. At birth, then, the dental pulps and their partially calcified crowns are completely enclosed in bony crypts. In any dry specimen in which this bony roof is found complete the external surfaces of the calcified crowns within the crypts are invariably found to lack the lustre and vitrified appearance one finds upon the surface of adult teeth. In a crypt from which the roof has been partially or wholly absorbed the tooth-crown is found to be completely calcified; from which we may, I think, infer that completion of the process of amelification is the necessary prelude to absorption of the roof of the crypt. At the time when this absorption occurs the bases of the crowns are in closer contact with the floors of the crypts in which they are contained than at any other time, but there always intervenes, of course, the vascular tissue which marks the junction of the tooth-sac and the pulp.

It is in the unfamiliar character as the active agent in the translation of the teeth from the crypts in which they originate to the position they finally occupy in the mouth that I wish you to make the acquaintance of the dental pulp to-day. To do this it was first necessary to remind you of its anatomical relations, which have been altogether ignored by the authors of the various theories which from time to time have been advanced to explain that interesting developmental process which we term "eruption of the teeth." Before stating the case for the dental pulp let us pause a moment to consider these theories.

One of the oldest of them is that the eruption of the teeth is due to the elongation of their roots. As long ago as 1835 Thomas Bell wrote: "As ossification proceeds, the roots of the teeth continue to elongate, until first those of the incisors, and subsequently the others, can no longer be contained within the alveoli." This theory is still very popular, but against it the following insuperable objections have been raised: First, that the distance travelled by the crown of the tooth is sometimes greater than the length of its root. Secondly, that teeth with comparatively little root sometimes erupt, whilst others with fully formed roots remain unerupted. Thirdly, that teeth with roots fully formed may remain unerupted for some length of time and then subsequently erupt.

The following quotation from an article by Dr. Pierce, entitled "The Eruption and Structural Relations of the Deciduous and Permanent Teeth," in the "American System of Dentistry" (1887), clearly shows that there are some writers who even yet regard this theory with favor. He says, "The absorption of the superimposed tissue from the advancing crown and the elongation or growth of the root by an increase in the pulpy mass or formative tissue and its calcification are the progressive developmental processes which we term 'eruption of the teeth.'

"The force by which the teeth are propelled towards and through the mucous surface into position is thought by many to be something in addition to that indicated above as the result of normal growth."

In explanation of the eruption of teeth with unformed roots,

Dr. Pierce writes, in the same article: "The question at once arises whether such premature presentation of the tooth-crown is not wholly due to an absorption or wasting of the superimposed tissue, rather than to the elevation of the crown, which could not well take place without the growth of root, unless it were from the contraction or an expulsive effort of the tooth follicle." The same writer offers the following ingenious explanation of the eruption of teeth with roots that were fully formed some time previously: "There is a mechanical force, however, acting on all such teeth, tending to bring them to the surface, the same as on an unantagonized tooth, inducing its elongation or protrusion from the socket. The repeated closing of the jaws must exert to a large extent this mechanical force, just as the bung in a barrel is elevated by a blow being struck upon the stave or either side of it."

Tomes's explanation of the same phenomenon is not quite so ingenious, but, as will appear later, is not less unsatisfactory. His account of the eruption of a canine tooth of a human female at the age of forty-five is as follows:

"Supposing it, then, to be admitted that the tooth was completely developed before the process of cutting commenced, the process in itself must be in some respects different from that which occurs when teeth are cut under ordinary circumstances. When the process is normal as respects time and the stage of development of the tooth, the crown appears through the gum long before the root has attained its full length. The crown is in great part brought towards the surface of the gum by the progressive lengthening of the root, and is afterwards still further raised by the same process. Now, when the eruption is accomplished subsequent to the development of the root, the movement of the tooth must be effected by some other means than by the progressive lengthening of the root. The completed tooth has to change its place without itself undergoing any change. The bone which stands in its way must be absorbed, and the lower portion of the socket from which the root of the tooth emerges must be contracted by the deposition of the bone. Indeed, in the absence of a better hypothesis it may be assumed that the gradual contraction of the socket is the means used by nature for bringing teeth to the surface when the process of eruption has been delayed beyond the normal period. In the one case the movement is effected by the development of bone within the alveolus, in the other by the progressive development and consequent lengthening of the tooth."

Another theory that for a time held sway was that of Delabarre. The following account of it from Harris's "Dental Surgery" (1863) is almost touching, and shows the fascination it exercised over lovers of analogy:

"The able physiologist and learned dentist, Delabarre, has advanced a most ingenious theory upon this subject. He believes that the passage of a tooth through the gum, or rather its escape from its crypt, is effected in precisely the same manner as is the birth of a child. He regards the sac attached above to the gum and below to the neck of the tooth as the chief agent in the eruption, and believes that it is by its contraction that the latter is raised from the bottom of the alveolus and ultimately forced through the dilated orifice of the capsule and gum. This is the most rational theory that has been advanced; it explains upon principles of sound physiology this most wonderful and curious operation of the economy."

This romantic theory lost favor chiefly because its most ardent supporters were unable to demonstrate the slightest resemblance between the dental sac and the female organ to which they imagined it was analogous. It would not have been included here were it not that a passage in one of the above quotations from the writings of Dr. Pierce indicates that the theory has not yet lost its pristine power.

It must have been the improbability of Delabarre's theory that encouraged Coleman to propound another, the acceptance of which would involve the abandonment of existing views as to the growth and development of the jaws. He imagined the teeth to be carried to the surface by a series of "bone currents," in other words, by interstitial growth of bone peculiar to the jaws, and then laid bare by the absorption of the alveolar margins. The reluctance displayed by our leading physiologists to lay aside their views as to the growth of bone, and to accept Coleman's, no doubt largely accounts for the modified enthusiasm with which his theory has been received. The greatest objection to it, perhaps, apart from this, is that while it accounts more or less satisfactorily for the eruption of the teeth, it fails to explain how it is that some teeth never do erupt. It is difficult to imagine a lateral or canine tooth stemming the tide of bone-currents that its immediate neighbors have found it impossible to resist.

Another theory is that the teeth are raised by a deposit of bone at the bottom of the crypts in which they are contained; and yet another (which is the last to be considered here) is that the teeth are forced into their destined places by contraction of the alveoli. Of these two theories, the latter would only account for the eruption of such teeth as have a single conical root, because in the case of teeth with two or more roots such contraction would tend to retard rather than assist eruption; while the former rests upon an assumption for which, as we shall presently see, there is absolutely no foundation.

I would here draw your attention to a diagram which represents a specimen in the Museum of the Odontological Society of Great Britain. It shows as clearly as possible that the distance of the bottom of the crypt containing the second molar from the interior dental canal is very little less than the distance of the apices of the roots of the first molar from the same landmark. It is clear, therefore, that the eruption of the first molar could not have been due to bone deposition upon the floor of its crypt. Reference to the same illustration will also convince you that narrowing of the alveoli containing the roots of the first molar would have retarded rather than assisted its eruption.

It appears to me that the chief objection to the root elongation and bone formation theories is a physiological one. It is extremely difficult to conceive such a process as dentine formation exercising independent mechanical force! But granting that it may be so, upon what structure is that force exercised? in other words, to put the matter clearly and concisely, if somewhat vulgarly, what does the root shove against?

Since the forming root is never in actual contact with its bony surroundings, it must necessarily be against the vascular material in which it is embedded. Now, this tissue appears post mortem of far too jelly-like a consistence to oppose any effective resistance by virtue of its own structure, and yet such resistance there must be or the tissue would be obliterated. Whence, then, are its resisting properties derived? Necessarily from the blood-pressure. Therefore, assuming that the physiological process of dentinification *can* exercise independent mechanical force and *is* a factor in the causation of eruption, it must, since action and reaction are equal and opposite, divide the honors with the blood-pressure—a factor hitherto quite unrecognized. Indeed, it is obvious that any

*vis a tergo* must act through the vascular material surrounding the root; and it follows that such force cannot be greater than the blood-pressure or it would cut off the blood-supply to the root.

But is a force other than the blood-pressure a necessary hypothesis when we consider the exceptionally advantageous conditions under which it acts? Let us illustrate these conditions by a diagram.

A, B, C, and D represent a section through a lower molar tooth and its crypt at various stages of development. In A it is obvious that the pulp forms a fleshy column of vascular tissue upon which the crown really rests. The pulp itself is injected by the force of the blood-pressure entering almost directly from A′, an artery of considerable size. Under the calcifying margins of the crown is the pad of tissue that forms the junction of the sac and pulp, and between the crown and the walls of the crypt is the vascular tooth-sac which is injected from the same source as the pulp. Above the crown is the oral mucous membrane and submucous tissue. Now it is obvious that the blood-pressure exerted in the pulp-tissue acts upon the crown at a considerable mechanical advantage in comparison with the pressure in the tissues overlying the crown. Indeed, it is only necessary to glance at the diagram to understand how it is that some teeth travel so quickly to their destined position when once their crowns have emerged from the gum. In fact, with regard to this point, it is a marvel that dentists who have many opportunities of observing the rapidity with which teeth sometimes move during eruption should ever have been induced to regard the comparatively slow process of dentinification as the active agent in the matter. As eruption proceeds, so does root formation, the former making space for the latter. Indeed, if I may be allowed to illustrate the process in a homely fashion and in the way it presents itself to me, the mechanism is somewhat similar to that employed by a sweep in cleaning a chimney. I do not know whether you, in this country, have improved upon our method of "chimney-sweeping," so perhaps I had better explain that our sweep employs a circular brush and a bundle of rods with screw joints. He places the brush in the chimney and screws in the first rod, then he pushes the brush up the chimney the length of the rod, and then screws in another rod, and so on. Now, the sweep represents the pulp, the force he uses to push the brush up the chimney represents the

Section through a lower molar tooth.

blood-pressure, and the screwing in of a fresh rod represents the various stages of root formation.

Referring again to the diagram, we notice that each stage in the formation of the root diminishes the extrusive tendency of the pulp, until when the root is complete it becomes practically *nil.* Moreover, after the tooth-crown has emerged from the gum the tooth-sac, which has now become the peridental membrane, forms a ligament for the tooth, being attached both to the roots and to the socket. The vascularity of this membrane still endows it, however, with an extrusive tendency which is in itself sufficient to account for the gradual elongation of unopposed teeth, and is probably the chief means by which the proper occlusion of opposing teeth is maintained. If we have any doubt, in the case of healthy teeth, as to whether this elongation is due to the normal blood-pressure exerted in the peridental membrane, they should surely be dispelled by the clinical phenomena that present themselves when the blood-pressure is pathologically augmented; in other words, when inflammation of the peridental membrane supervenes. When we consider the mechanical conditions illustrated by the foregoing diagram in conjunction with the pulsating and expansive force exercised by the blood-pressure, does not the necessity for another eruptive force disappear and Dr. Pierce's bung-and-barrel explanation of the elongation of unopposed teeth become somewhat superfluous?

If we assume that the blood-pressure, acting in the manner above described, is the sole active mechanical factor in determining the eruption of the teeth, will it account for the phenomena other theories fail to explain? That the crown of the tooth sometimes travels a distance greater than the length of its root, that teeth sometimes erupt subsequently to the formation of their roots, and that teeth with comparatively little root occasionally erupt are facts all in favor of such hypothesis, and can be explained by it.

The fact that teeth with fully formed roots remain unerupted can be more easily explained by this theory than by any other, because it alone can account for the space obtained for the fully developed roots which often occupy abnormal positions. In other words, the blood-pressure acting as it does equally in all directions makes room for the developing root in the direction of least resistance. Normally this is in the direction of the advancing crown, but occasionally it is elsewhere. The continuous eruption of teeth with persistent pulps, which neither Delabarre's nor Coleman's theory could possibly explain, is a very simple problem if we admit the blood-pressure as the active mechanical factor.

I am therefore of opinion that upon anatomical and physiological grounds alone are we justified in assuming that *the blood pressure exerted in the vascular tissue which lies between a developing tooth and its bony surroundings is the active mechanical factor in the process known as the eruption of the teeth.*

So far the purely mechanical aspect of the question has been alone considered; but if your patience will endure the strain, there are one or two points of physiological interest that have so direct a bearing upon the subject that it would be as well to include them here.

Some years ago I recorded the observation that, in young people, when a back tooth had lost its antagonists, the characteristic elongation which takes place under those circumstances varied in cases in which the pulps of the unopposing teeth were dead from those in which they were living. In the former instance, although the elongation of the teeth took place, it was unaccompanied by any downgrowth (or, in the case of a lower tooth, upgrowth) of the alveolar ridge; whereas, in the latter case there was a corresponding deepening of the alveolar ridge. In other words, in the case of dead teeth there was simply extrusion from the alveolus, but in the case of living there was growth of the alveolar ridge. It appears obvious, therefore, that the growth of the alveolar process is dependent upon the integrity of the dental pulp; or, in other words, that the pulps of the teeth as a whole exercise a trophic influence with regard to the alveolar process. I am of opinion that extirpation of the pulp of a tooth causes a marked and permanent alteration in the vascular condition of the peridental membrane; in fact, a disturbance of vasomotor equilibrium in the direction of a paralysis of the vasoconstrictor mechanism.

The foregoing remarks apply to both the permanent and temporary dentitions; but the pulps of the temporary teeth exercise another kind of trophic influence which seems to have escaped the notice of dental writers,—namely, their influence upon the process of resorption of the roots of the temporary teeth. In the case of temporary teeth in which the pulps are destroyed at the time when resorption of the roots should commence, resorption, strictly so-called, does not occur at all. In such cases a certain amount of absorption of the root, as a rule, takes place, just as it often does in the case of dead permanent teeth, the macroscopic appearance of the roots in both cases being strikingly similar. This absorption is a pathological process, and differs markedly from the physiological process of resorption. It is a much slower process, and that is one reason why we so frequently find the apices of the roots of dead temporary teeth protruding from the labial or buccal surfaces of the alveolar ridges, causing that ulceration of the mucous membrane of the cheeks or lips with which we are so familiar in the case of children whose milk teeth have been neglected.

The explanation of this common phenomenon is simple. The death of the pulp of the temporary tooth has left its root incapable of resorption and its socket prone to degeneration. Absorption is too slow a process to make room for the crown of the permanent successor, which soon impinges upon the dead root, deflects it, and thrusts its apex through the degenerated alveolar process and the superjacent soft tissues. In those cases in which death of the temporary tooth has taken place some time after the process of resorption has commenced, and the root is, in consequence, shortened, the pressure of the advancing permanent tooth simply tilts the root until it takes a nearly horizontal position, the crown, if any remain, being correspondingly deflected. Other phenomena which admit of a similar explanation are familiar to us all, and need not be enumerated.

From the time when the dental pulp is "nothing more than a part of the mesoblastic myxomatous tissue of the jaw, which has become more rich in vessels and cells than the other neighboring part," up to the time when commencing senile degeneration presages the termination of its physiological activity, it is one of the busiest exponents of local government observable in the whole domain of human physiology. While it is hard at work constructing the tooth, it regulates the blood-pressure that causes that organ to travel to its appointed place in the mouth, at the same time building up the bony walls that enable that pressure to act at a mechanical advantage. Then, in the case of the temporary teeth, it superintends the demolition of the very structure it has been at such pains to create; and finally, in the case of the permanent teeth it controls the nutrition of those parts upon the integrity of which the tooth is dependent for the proper exercise of its function.

## Figures and Tables

**Figure f1:**